# Transient, Isolated Head Tremor in “Unaffected” Individuals: Is Essential Tremor an Even More Prevalent Disease Than We Suppose?

**DOI:** 10.3389/fneur.2018.00570

**Published:** 2018-07-13

**Authors:** Elan D. Louis, James H. Meyers, Ashley D. Cristal, Amar Patel, Sule Tinaz, Seth L. Pullman, Lorraine N. Clark, Ruth Ottman, Pam Factor-Litvak

**Affiliations:** ^1^Department of Neurology, Yale School of Medicine, Yale University, New Haven, CT, United States; ^2^Department of Chronic Disease Epidemiology, Yale School of Public Health, Yale University, New Haven, CT, United States; ^3^Center for Neuroepidemiology and Clinical Neurological Research, Yale School of Medicine, Yale University, New Haven, CT, United States; ^4^Department of Neurology, College of Physicians and Surgeons, Columbia University, New York, NY, United States; ^5^Taub Institute for Research on Alzheimer's Disease and the Aging Brain, College of Physicians and Surgeons, Columbia University, New York, NY, United States; ^6^Department of Pathology and Cell Biology, Columbia University Medical Center, New York, NY, United States; ^7^G.H. Sergievsky Center, College of Physicians and Surgeons, Columbia University, New York, NY, United States; ^8^Department of Epidemiology, Mailman School of Public Health, Columbia University, New York, NY, United States; ^9^Division of Epidemiology, New York State Psychiatric Institute, New York, NY, United States

**Keywords:** essential tremor, head tremor, clinical, genetics, epidemiology, prevalence, diagnosis, diagnostic misclassification

## Abstract

**Background:** Mild and transient head tremor may sometimes be observed in otherwise tremor-free relatives of essential tremor (ET) cases, although its prevalence is unclear. A diagnostic question is whether this transient, isolated head tremor, often observed as no more than a wobble, is an early manifestation of ET or whether it is a normal finding. A direct comparison with controls is needed.

**Methods:** Two hundred and forty-one first-degree relatives of ET cases (FD-ET) and 77 spousal controls (Co) were enrolled in a study of ET. Each underwent a detailed evaluation that included a tremor history and videotaped neurological examination. None of the enrollees reported tremor, had a prior diagnosis of ET, or had significant tremor on screening spirals. All videotaped examinations were initially reviewed by a movement disorder neurologist blinded to subject type, and among those with head tremor on examination, co-reviewed by two additional movement disorders neurologists.

**Results:** Twenty-six (10.8, 95% Confidence interval [CI] = 7.5–15.3%) of 241 FD-ET vs. 2 (2.6, 95% CI = 0.7–9.0%) of 77 Co had isolated, transient head tremor (odds ratio = 4.54, 95% CI = 1.05–19.57, p = 0.04). No enrollee had significant upper extremity tremor and none met inclusion criteria for ET based on the presence of upper extremity tremor. With one exception, head tremor occurred during or after phonation. It was always transient (generally a single back and forth wobble) and rare (observed briefly on one or two occasions during the videotaped examination) and had a faster frequency, lower amplitude and a different quality than voluntary head shaking.

**Conclusion:** The basis for the observed isolated head tremor is unknown, but it could be an early feature of ET in ET families.Indeed, one-in-ten otherwise unaffected first-degree relatives of ET cases exhibited such tremor. To a far lesser extent it was also observed in “unaffected” controls. In both, it is likely a sign of early, emerging, undiagnosed ET, although follow-up studies are needed to confirm this. If it were ET, it would indicate that the prevalence of ET may be considerably higher than previously suspected.

## Introduction

Essential tremor (ET) is the most common tremor disorder and among the most prevalent movement disorders (1, 2). In a meta-analysis of population-based studies, the prevalence among individuals aged 65 and older was 4.6% (1). In a descriptive analysis of the same studies, the median crude prevalence was 6.3% in this age group (1). Prevalence increases with age (1, 3–9), and in one study, the crude prevalence reached 21.7% in persons aged 95 and older (1, 10).

We previously reported that speech and certain vocal tasks can trigger a transient head tremor in ET cases (11). In a single study of two small series of asymptomatic relatives of ET cases who had normal, physiological levels of arm tremor on examination, 1/26 (3.7%) and 6/27 (23.1%) evidenced a transient head wobble on examination during or directly after sustained phonation, speech, or reading aloud (12). Given these widely disparate results, our current estimate of the prevalence of this condition is imprecise (i.e., somewhere between 3.7 and 23.1%) (12). This is likely a function of the small sample sizes in those series (*n* = 26 and 27) (12); hence, larger samples are needed. Also, as the above study did not enroll controls (12), an unresolved diagnostic issue is whether transient head wobble is an early manifestation of ET or whether it is a normal finding.

In the current study, we enrolled 241 self-reportedly “unaffected” relatives of ET cases, which is a sample that is ~10 times greater than in prior series (12), as well as a control sample of 77 individuals (i.e., the spouses of unaffected relatives). In addition to the expanded sample size and the enrollment of controls, the assessment of head tremor on neurological examination was more detailed than in our prior study (12). Furthermore, all videotaped examinations were initially reviewed by a movement disorder neurologist blinded to subject type, and among those with head tremor on examination, co-reviewed by two additional movement disorders neurologists.

Our goals were several. First, to assess the prevalence and clinical features of isolated head tremor in “unaffected” first-degree relatives of ET cases and their spousal controls. The large sample size facilitated a more precise estimate of its prevalence than in the prior study (12). Second, to evaluate whether this isolated head tremor is merely a normal finding or conversely an early-ET feature. A higher prevalence in first-degree relatives of ET cases than their spouses would suggest that the finding is ET-related (i.e., not normal). Third, to examine the clinical correlates (e.g., age, gender) of isolated head tremor. Fourth, to describe the tremor phenomenology.

From a public health perspective, the aim of this report is to evaluate whether this subtle and interesting clinical finding should make us revise-upwards our estimates of the prevalence of ET, a disease that is already considered highly prevalent. From a clinical research perspective, the goal is to provide researchers with guidance as to how to classify these individuals both in genetic and epidemiological studies —are they normal or do they have emerging ET? If this issue were to be handled incorrectly or ignored, investigators could be introducing considerable diagnostic misclassification into their studies.

## Methods

### Introduction

FD-ET and their spouses (i.e., Co) were screened for enrollment in an ongoing environmental epidemiological study of ET (May 2016–present) (12, 13).

ET cases had been ascertained from study advertisements to members of the International Essential Tremor Foundation, current ET research studies at Yale University, and the clinical practice of the Yale Movement Disorders Group (12, 13).

### Screening process for unaffected FD-ET

The screening process for unaffected FD-ET was as follows. First, ET cases informed the study staff of all reportedly unaffected living first-degree relatives age ≥40 years. With permission, the study staff contacted these family members by telephone. During this telephone call, they were consented using a protocol approved by the Yale University Institutional Review Board and then interviewed. During the interview, a 12–item tremor screening questionnaire (14) was administered during which they were asked whether they carried a diagnosis of ET. They also completed and mailed four hand-drawn spirals (two right, two left), which were rated by a senior movement disorder neurologist (E.D.L.) using the following scale: 0, 0.5, 1, 1.5, 2, and 3 (see definitions and examples in Louis et al.) (15).

We initially categorized these FD-ET as unaffected if they met each of the following criteria: (1) they did not report tremor during the 12–item telephone-administered tremor screening questionnaire (14), (2) they had never been assigned an ET diagnosis by a treating physician, and (3) each of their four screening spirals were assigned a rating <2.0 (i.e., no tremor or mild tremor).

### In-person clinical evaluation of FD-ET

If initially categorized as unaffected, FD-ETs were invited for an in-person clinical evaluation that was conducted by study staff in the FD-ETs' homes and included several clinical questionnaires, which elicited data on demographics, tremor features, medical history, and medications (13). Also, family history data (i.e., data on reportedly affected relatives) were elicited. The number of reportedly affected first-degree relatives was defined as the genetic load, and was a simple measure of the extent to which ET ran in the family of each subject. The Cumulative Illness Rating Scale (CIRS) (range = 0–42 [maximum co-morbidity]) (16), a measure of medical co-morbidity, was administered; this assessed the presence and severity of illnesses (none = 0, mild = 1, moderate = 2, severe = 3) in 14 body systems.

As in our previous studies (12, 13), each enrollee underwent a 20–30 min standardized videotaped neurological examination, which included a detailed assessment of postural tremor, five tests for kinetic tremor, the motor portion of the Unified Parkinson's Disease Rating Scale (17) excluding an assessment of rigidity, and a comprehensive assessment of dystonia (12, 13). The examination also included a detailed assessment of head, jaw, and voice tremors. For head tremor, enrollees first were assessed while seated quietly and facing the camera, during brief conversational speech, during sustained phonation (“ahh” and “eee” for 10–15 s each, and then repeated) and while reading a standard passage from a sheet of paper. Hence, the evaluation was lengthier than in our prior study because we repeated tasks (12). Head tremor was also potentially detectable during much of the remainder of the 20–30 min videotaped assessment (e.g., while drinking water from a cup, while using a spoon, while touching finger-to-nose) (12). E.D.L. reviewed all videotaped examinations. The severity of postural and kinetic tremors in the upper extremities was rated (0–3), resulting in a total tremor score (range 0–36, maximum), a measure of the severity of the action tremor. Head tremor was coded as present or absent and was distinguished from dystonic tremor by the absence of twisting or tilting movements of the neck, jerk-like or sustained neck deviation, or hypertrophy of neck muscles (18). Head tremor had to be both rhythmic and oscillatory to be ascribed to ET rather than dystonia (18). In those participants with head tremor coded as present on examination by E.D.L., regardless of its severity, the videotaped head tremor was co-reviewed by two additional movement disorders neurologists (A.P., S.T.) to assess for features of dystonia, and final decision (ET-like head tremor vs. head tremor with dystonic features) was assigned based on consensus of the three (E.D.L., A.P., S.T.).

FD-ET were re-evaluated for a potential ET diagnosis based on review of questionnaires and videotaped neurological examination data (13). Diagnoses of ET were assigned based on published diagnostic criteria (moderate or greater amplitude kinetic tremor during three or more activities or a clear head tremor in the absence of PD or another known cause; e.g., medication-induced tremor, tremor from hyperthyroidism) (19–21).

### Final inclusion of FD-ET

FD-ET were included in these analyses if they were initially categorized as unaffected (see above) and they were NOT diagnosed with ET based on the in-person evaluation. Of note is that we included the individuals with isolated, transient head tremor who are the focal point of this study as the diagnostic significance of their head tremor was of uncertain significance.

### Parallel procedure for screening and evaluating Co

Co were also screened, if available. Each then underwent the same screening process, in-person questionnaire, and videotaped examination. They were included in these analyses if (1) they were initially categorized as unaffected, (2) reported no family history of ET, and (3) they were NOT diagnosed with ET based on the in-person evaluation. Of note is that we included the individuals with isolated, transient head tremor who are the focal point of this study as the diagnostic significance of their head tremor was of uncertain significance.

### Final sample

To date, we have screened and enrolled 467 individuals. We excluded 2 Co with unclear family histories; 465 enrollees remained. Of these, 322 were either categorized as unaffected (*n* = 294) or had isolated head tremor (*n* = 28). We further excluded 4 enrollees with incomplete data. Hence, the final sample was 318 (241 FD-ET and 77 Co).

### Study power

The prevalence of isolated head tremor in FD-ET was 3.7% in one series and 23.1% in another (mean = 13.4%) (12). In Co, it was expected to be close to zero. Our sample size of enrollees (241 FD-ET and 77 Co) provides 99.8% power to detect a difference between FD-ET and Co in the prevalence of isolated head tremor (assuming prevalence = 13.4% in FD-ET and 1.0% in Co, and alpha = 0.05 and two-sided test).

### Statistical analyses

For continuous variables (e.g., age, education), normality was assessed using a Kolmogorov–Smirnov test; when the distribution was not normal (p < 0.05), non-parametric tests were used (e.g., Mann–Whitney test). We compared FD-ET to Co in terms of demographic and clinical features (Table 1) using Mann Whitney tests, chi-square tests, and Fisher's exact tests. We performed similar analyses, comparing enrollees with isolated head tremor to enrollees without isolated head tremor (Table 2). We reported the prevalence of isolated head tremor in each of our two groups (FD-ET and Co), and in a logistic regression, calculated the odds of isolated head tremor in FD-ET compared to Co, thereby yielding odds ratios (ORs) and 95% confidence intervals (CI). In a stratified analysis, we considered the confounding effects of gender on the prevalence of isolated head tremor in each group. Finally, we examined whether FD-ET with isolated head tremor differed from FD-ET without isolated head tremor in terms of their genetic load or total tremor score (Mann–Whitney tests).

**Table 1:** Demographic and clinical features of 241 FD-ET vs. 77 Co.

**Demographic or clinical feature**	**FD-ET *n* = 241**	**Co *n* = 77**	**Significance**
Current age in years	57.2 ± 9.9 [56.0]	56.9 ± 9.5 [57.0]	0.90^a^
Female gender	164 (68.0)	25 (32.5)	<0.001^b^
Education in years	16.7 ± 2.7 [16.0]	16.5 ± 3.5 [16.0]	0.66^a^
White race	232 (96.3)	73 (94.8)	0.52^c^
Current cigarette smoker	4 (1.7)	0 (0.0)	0.58^c^
Number of prescription medications	2.1 ± 2.6 [1.0]	2.6 ± 2.7 [2.0]	0.20^a^
CIRS score	4.4 ± 3.5 [4.0]	4.0 ± 3.6 [3.0]	0.28^a^
Genetic load^d^	1.4 ± 0.9 [1.0]	0.0 ± 0.0 [0.0]	<0.001^a^
Total tremor score	7.4 ± 2.7 [7.0]	6.4 ± 2.9 [5.75]	0.002^a^

*Values are mean ± standard deviation [median] or number (percent). CIRS, Cumulative Illness Rating Scale*.

a *Mann–Whitney test*.

b *Chi-square test*.

c *Fisher's exact test*.

d *The number of reportedly affected first-degree relatives was defined as the genetic load*.

**Table 2:** Demographic and clinical features of enrollees with vs. without isolated head tremor.

	**FD-ET (*****n*** = **241)**			**Co (*****n*** = **77)**		
**Demographic or clinical feature**	**Isolated head tremor present (*n* = 26)**	**Isolated head tremor absent (*n* = 215)**	**Sig**	**Isolated head tremor present (*n* = 2)**	**Isolated head tremor absent (*n* = 75)**	**Sig**
Current age in years	60.6 ± 10.3 [62.5]	56.8 ± 9.8 [56.0]	0.05^a^	52.5 ± 16.3 [52.5]	57.0 ± 9.4 [57.0]	0.69^a^
Female gender	22 (84.6)	142 (66.0)	0.055^b^	1 (50.0)	24 (32.0)	0.55^c^
Education in years	16.2 ± 1.9 [16.0]	16.7 ± 2.8 [16.0]	0.28^a^	17.5 ± 2.1 [17.5]	16.5 ± 3.5 [16.0]	0.50^a^
White race	26 (100)	206 (95.8)	0.60^c^	1 (50.0)	72 (96.0)	0.10^c^
Current cigarette smoker	0 (0.0)	4 (1.9)	1.00^c^	0 (0.0)	0 (0.0)	1.00^c^
Number of prescription medications	3.2 ± 4.5 [1.5]	1.9 ± 2.2 [1.0]	0.47^a^	2.0 ± 1.4 [2.0]	2.6 ± 2.8 [2.0]	0.99^a^
CIRS score	5.4 ± 4.4 [4.0]	4.3 ± 3.3 [4.0]	0.31^a^	1.5 ± 0.7 [1.5]	4.0 ± 3.6 [3.0]	0.25^a^
Genetic load^d^	1.3 ± 0.6 [1.0]	1.4 ± 0.9 [1.0]	0.85^a^	0.0 ± 0.0 [0.0]	0.0 ± 0.0 [0.0]	1.0^a^
Total tremor score	7.6 ± 2.8 [7.0]	7.4 ± 2.7 [7.0]	0.87^a^	4.75 ± 1.1 [4.75]	6.5 ± 2.9 [6.0]	0.42^a^

Values are mean ± standard deviation [median] or number (percent). CIRS, Cumulative Illness Rating Scale; Sig, significance.

a Mann–Whitney test.

b Chi-square test.

c Fisher's exact test.

d The number of reportedly affected first-degree relatives was defined as the genetic load.

## Results

### Characteristics of enrollees

There were 318 enrollees (241 FD-ET and 77 Co). FD-ET and Co were similar in age and most other demographic factors (e.g., education, race, cigarette smoker); however, they differed by gender (p < 0.001, Table 1). As expected, the genetic load (i.e., the number of reportedly affected first-degree relatives) was higher in the FD-ET group (p < 0.001). The total tremor score was also higher in the FD-ET group (p = 0.002, Table 1), although tremor scores were uniformly low in both groups and none met inclusion criteria for ET based on the presence of upper extremity tremor. The two groups were similar with respect to number of prescription medications and CIRS scores (Table 1).

### Prevalence and clinical features of isolated head tremor

Twenty-six (10.8, 95% CI = 7.5–15.3%) of 241 FD-ET vs. 2 (2.6, 95% CI = 0.7–9.0%) of 77 Co had isolated, transient head tremor with no dystonic features (χ^2^ = 4.88, p = 0.027). The odds of having isolated head tremor was 4.5 times greater in FD-ET than Co (OR = 4.54, 95% CI = 1.05–19.57, p = 0.04). With only one exception, the head tremor occurred during or after phonation. The tremor was always very transient (generally a single back and forth wobble observed) and rare (observed on one or two occasions during the videotape) and had a faster frequency, lower amplitude and a different quality than voluntary head shaking (e.g., when a subject gestures “no” with their head). For several examples of these types of head tremors, see videotape in Louis et al. (12). In addition to the 26 FD-ET with isolated head tremor noted above, there were 3 additional FD-ET and 0 controls who had head tremor with dystonic features.

### Clinical correlates of isolated head tremor

We compared enrollees with isolated head tremor to those who did not have isolated head tremor (Table 2). Among FD-ET, enrollees with isolated head tremor were marginally older and a larger proportion were women. Indeed, the large majority (22/26, 84.6%) of FD-ET with isolated head tremor were women. Among Co, those with isolated head tremor were more likely to be women, although the small number with head tremor (n = 2) limited the value of statistical comparisons.

FD-ET and Co differed with respect to gender. Therefore, we stratified by gender. In women, 22/164 (33.5%) FD-ET vs. 1/25 (4.0%) Co had isolated head tremor. In men, 4/77 (5.2%) FD-ET vs. 1/52 (1.9%) Co had isolated head tremor. In men, the odds of having isolated head tremor was 2.8 times greater in FD-ET than Co (OR = 2.80, 95% CI = 0.30–25.74), although given the small sample size in these stratified analyses, the difference was not significant (p = 0.36). In women, the odds of having isolated head tremor was 3.72 times greater in FD-ET than Co (OR = 3.72, 95% CI = 0.48–28.89), although given the small sample size in these stratified analyses, the difference was not significant (p = 0.21).

FD-ET with isolated head tremor did not differ from FD-ET without isolated head tremor in terms of their genetic load (1.3 ± 0.6 [median = 1.0] vs. 1.4 ± 0.9 [median = 1.0], Mann Whitney test = 0.20, *p* = 0.85) or total tremor score (7.6 ± 2.8 [median = 7.0] vs. 7.4 ± 2.7 [median = 7.0], Mann Whitney test = 0.16 *p* = 0.87).

## Discussion

We enrolled a large sample of self-reportedly “unaffected” relatives of ET cases as well as a control sample. Twenty-six (10.8, 95% CI = 7.5–15.3%) of 241 FD-ET had isolated head tremor. In an earlier report, the number of relatives of cases with head tremor was small (i.e., 6 in one series and 1 in the other) and the total number of relatives was similarly small (26 in one series and 27 in the other) (12). Hence, estimates of the prevalence of this condition varied widely— as low as 3.7% (95% CI = 0.7–18.3%) in one series and as high as 23.1% (95% CI = 11.0–42.1%) in the other (12). The current series, with a larger sample, provides a more stable estimate, with far narrower confidence intervals, and with a point estimate (10.8%) that lies between the two extremes of the prior study (3.7 and 23.1%), although closer to the more conservative estimate. The point estimate indicates that one-in-ten reportedly “unaffected” screened first-degree relatives of ET cases could indeed have early ET.

The issue is whether this head tremor we observed is a normal finding or a manifestation of ET. First, the 4.5-fold increased odds of having isolated head tremor in FD-ET than Co (OR = 4.54, 95% CI = 1.05–19.57, p = 0.04), suggests that this is a manifestation of ET. Second, although 2.6% of Co had the finding, this could easily be undiagnosed, early ET, and is consistent with prevalence estimates for undiagnosed sporadic ET (1, 22, 23). Third, although some degree of upper limb tremor can be a normal finding (24–26), as can voice tremor under stressed conditions, current thinking is that head tremor, even if very transient, is not a normal finding (27). The current study was not a longitudinal one. Following the clinical progression of the subjects with isolated head tremor would ultimately answer the question as to whether they developed more manifest ET over time.

Another possibility that should be carefully considered is whether the isolated head tremor was simply due to dystonia. Patients with dystonia may certainly manifest with isolated head tremor (28), making this a distinct possibility. However, we think this is a less likely possibility than ET for several reasons. First, when isolated head tremor was noted on videotaped examination, two additional movement disorder neurologists co-reviewed the videotapes. During this review, head tremor of ET was carefully distinguished from dystonic tremor by the absence of twisting or tilting movements of the neck, jerk-like or sustained neck deviation, or hypertrophy of neck muscles (18). Head tremor had to be both rhythmic and oscillatory to be ascribed to ET rather than dystonia (18). Indeed, in this manner, we identified three additional FD-ET with isolated head tremor that we attributed to dystonia. Second, while it is still possible that some number of the 26 isolated head tremor cases may have had cervical dystonia/dystonic tremor in the complete absence of (1) dystonic neck postures, (2) neck deviation, (3) jerk-like neck movements, (4) hypertrophy of neck muscles, (5) non-rhythmicity of tremor, and (6) non-oscillatory tremor, the prevalence of dystonia in the population is several orders of magnitude less than that of ET (29); the prevalence of focal cervical dystonia is generally on the order of 0.002–0.02%, which is 200–2,000 less prevalent than ET among those age 40 and older (23). Hence, it is far more likely that the head tremor observed (esp. in the Co but also in the FD-ET) was due to ET than dystonia. Third, although ET and dystonia may co-occur in the same family, the families that we included in this study were ET families and they did not report family history of individuals with dystonia.

Several phenomenological points merit discussion. First, is the movement involuntary? We think the movement is clearly involuntary. On visual inspection, it has a faster frequency, lower amplitude and a different quality than voluntary head shaking (e.g., when a subject gestures “no” with their head). Furthermore, the movement is strikingly similar across subjects, further suggesting it is involuntary rather than voluntary. A second phenomenological point is that, with one exception, the head tremor always occurred during or after phonation. The same was true in our prior series—phonation seemed to release the phenomenon (11, 12). Thus, a voluntary movement seems to unmask or release an involuntary movement. This is sometimes also observed in patients with Parkinson's disease, among whom voluntary repetitive movements with one hand may unmask rest tremor in the contralateral, resting hand.

We examined the clinical correlates of isolated head tremor in FD-ET—those with isolated head tremor were marginally older than those without it, and a larger proportion were women. Indeed, the large majority (22/26, 84.6%) with isolated head tremor were women. Gender (30–32) and age (33, 34) have been associated with increased odds of head tremor in ET, serving as an additional indicator that the head tremor is likely a manifestation of ET rather than a normal finding; indeed, in prior cohorts, 78.4–88.1% of ET cases with head tremor were women (31, 32).

This study reports the results of granular phenotyping in study subjects, with (1) a lengthy and detailed examination (2) an examination that was recorded using high-definition (i.e., digital) recording and (3) an examination that was reviewed by a senior movement disorder neurologist with special interest in tremor. This enabled us to capture subtle findings. One epidemiological question is, what effect does our finding have on prevalence estimates of ET? The possibility that an additional 10.8% of first-degree relatives of ET cases also have subtle ET suggests that the prevalence of ET may be higher than currently suspected. But the real issue is the controls, as these are many magnitudes more prevalent in the population than first-degree relatives of ET cases. We found that 2.6% of normal controls, with normal amounts of upper limb tremor, had transient, isolated head tremor. This suggests that the prevalence of ET may be considerably higher than previously suspected. The mean age of the subjects in our study was ~57 years. In published reports, the reported prevalence of ET in this age group is on the order of 3–4% (1); adding an additional 2.6% (and therefore raising the value to 5.6 or 6.6%) would represent a 60–80% relative increase.

This study had certain limitations. Study subjects were enrolled within the framework of a single study; further studies of additional cohorts would be valuable. Second, head tremor was evaluated clinically rather than electrophysiologically, although the small number of oscillations in these cases would make that difficult. The fact that the in-person assessments were performed in subjects' homes did not make this feasible. Third, the number of Co was <100, and was far lower than that of FD-ET. Despite this low number, we were able to detect a significant difference in the odds of isolated head tremor between the two groups (*p* = 0.04), and this was the primary aim of the study. Nonetheless, the number of Co with isolated head tremor was small (*n* = 2); hence, assessing the clinical correlates of this tremor among Co was difficult, with small numbers in all cells (Table 2). Finally, the study design was cross-sectional rather than longitudinal. Following the clinical progression of the subjects with isolated head tremor would be of value to assess whether they developed more manifest ET over time. Unfortunately, the current study is not funded to do so. Strengths of the study included (1) the large sample size, (2) the detailed, uniform and prospective in-person assessment, (3) the detailed assessment of head tremor, which was more detailed than in our prior studies (11, 33) (4) the videotaping of the neurological examination, which allowed subtle findings to be reviewed and re-reviewed as many times as needed, (5) the review of a videotape by a senior movement disorders neurologist, and (6) the confirmation of findings by two additional movement disorder neurologists.

In summary, the basis for the observed isolated head tremor is unknown, but it could be an early feature of ET. Indeed, one-in-ten otherwise unaffected first-degree relatives of ET cases exhibited such tremor. It was also observed in 2–3% of “unaffected” controls. In both, it is likely a sign of early, emerging, undiagnosed ET, although follow-up studies are needed to confirm this. If it were ET, it would indicate that the prevalence of ET may be considerably higher than previously suspected. As individuals with such tremor not-infrequently are enrolled in genetic and epidemiological studies, investigators should be aware of the need to identify subtle forms of tremor in some study subjects and to properly classify these subjects as having emerging ET.

## Ethics statement

This study was carried out in accordance with the recommendations of the Yale University Institutional Review Board. The protocol was approved by the Yale University Institutional Review Board. All subjects gave written informed consent in accordance with the Declaration of Helsinki.

## Author contributions

EL was involved in the conception and design of this work, the statistical analysis and interpretation of data, the drafting of the manuscript, and gives final approval of this version to be published and agreement to be accountable for all aspects of the work in question. JM, AC, AP, and ST was involved in the acquisition of data and the critical revision of the manuscript, and gives final approval of the version to be published and agreement to be accountable for all aspects of the work in question. SP was involved in the conception and design of the work and the critical revision of the manuscript, and gives final approval of the version to be published and agreement to be accountable for all aspects of the work in question. LC, RO, and PF-L was involved with the conception and design of this work and the critical revision of the manuscript, and gives final approval of the version to be published and agreement to be accountable for all aspects of the work in question.

### Conflict of interest statement

The authors declare that the research was conducted in the absence of any commercial or financial relationships that could be construed as a potential conflict of interest.
